# Development and clinical validation of a novel detection kit for α-thalassemia in southern Chinese

**DOI:** 10.3389/fgene.2024.1457248

**Published:** 2024-09-05

**Authors:** Yi-Yuan Ge, Jun Xie, Yu-Wei Liao, Long-Xu Xie, Li-Ye Yang

**Affiliations:** ^1^ Guangzhou Hybribio Medicine Science and Technology Corporation, Guangzhou, Guangdong Province, China; ^2^ Precision Medical Lab Center, People’s Hospital of Yangjiang, Yangjiang, Guangdong Province, China

**Keywords:** reverse dot blot (RDB), α-thalassemia, polymerase chain reaction (PCR), China, ααα^anti−4.2^, ααα^anti−3.7^

## Abstract

**Objective:**

This study aimed to develop and assess a novel reverse dot blot assay for the simultaneous detection of 10 types of α-thalassemia alleles in the Chinese population, including six common variants of–^SEA^, -α^3.7^, -α^4.2^, α^CS^, α^QS^, and α^WS^, and four rare variants of ααα^anti−4.2^, ααα^anti−3.7^, ^--FIL^ deletion and^--THAI^ deletion.

**Methods:**

The novel thalassemia gene assay utilized a two-tier multiplex polymerase chain reaction amplification system and one round of hybridization. Genomic DNA samples were sourced from three hospitals in southern China. Each clinically validated DNA sample was re-evaluated using the new multiplex polymerase chain reaction/reverse dot blot assay Ⅲ (M-PCR/RDB Ⅲ).

**Results:**

The study analyzed a total of 1,148 unrelated participants, consisting of 810 thalassemia patients and 338 healthy control subjects. Valid hybridization results were obtained for 1,147 samples, with one case (thalassemia carrier) being excluded from the study due to the poor quality of DNA. All 1,147 samples, including those with α heterozygous thalassemia, α homozygous thalassemia, α compound heterozygous thalassemia, and control subjects were accurately genotyped, showing 100% concordance with the reference assays.

**Conclusion:**

The novel M-PCR/RDB Ⅲ assay proved to be simple, rapid, and precise, indicating its potential for genetic screening and clinical diagnosis of both common and rare α-thalassemia variants in Chinese populations.

## 1 Introduction

Thalassemia syndrome stands as the most prevalent single gene mutation disorder among humans (Weatherall.,1997; Taher et al., 2018). It encompasses two primary forms, namely α-thalassemia and β-thalassemia. β-thalassemia and α-thalassemia are inherited blood disorders characterized by reduced production of hemoglobin, the protein in red blood cells that carries oxygen throughout the body. β-thalassemia results from mutations in the *HBB* gene affecting β-globin, while α-thalassemia is caused by deletions or mutations in the *HBA1* and *HBA2* genes affecting α-globin, resulting in diminished or absent synthesis of the α-globin chain of hemoglobin (Hb) (Chen et al.,2002; Muncie and Campbell, 2009). These conditions manifest in varying degrees of anemia, with clinical severity ranging from mild to life-threatening. Understanding the genetic basis, types, clinical manifestations, diagnostic methods, and treatment options for both beta-thalassemia and alpha-thalassemia is crucial for effective management and care.

The prevalence of thalassemia is notably high in tropical and subtropical regions such as the Mediterranean basin, Africa, the Middle East, the Indian subcontinent, and Southeast Asia (Weatherall and Clegg, 2001; Viprakasit and Ekwattanakit,2018). Previous research has highlighted a marked incidence of thalassemia in southern China, particularly in the provinces of Guangxi, Guangdong, and Hainan (Xu et al.,2004; Xiong et al.,2010; Wang et al.,2023). In southern China, the^--SEA^ deletion ranks as the most prevalent mutation in α-thalassemia, followed by -α^3.7^ and -α^4.2^ (Xu et al.,2004; Xiong et al.,2010; Lin, M et al.,2013; Liang et al.,2023).

Various techniques, including reverse dot blot and gap-polymerase chain reaction (gap-PCR), have been utilized in thalassemia screening (Xu et al.,2004; Xiong et al.,2010; Lin et al.,2013; Liang et al., 2023). Yet, these methods are known for their high costs and labor-intensive procedures (Lin et al., 2013; Liang et al.,2023). Our prior studies introduced two multiplex PCR/reverse dot blot assays (M-PCR/RDB I and M-PCR/RDB II) that have become widely adopted in clinical thalassemia diagnosis in southern China (Lin et al., 2012; Liang et al., 2022). Despite their effectiveness, these assays were unable to distinguish relatively rare α variants, such as ααα^anti−4.2^, ααα^anti−3.7^, ^--FIL^ deletion, and^--THAI^ deletion.

This investigation presents a novel α-thalassemia genetic assay that employs a two-tier multiplex polymerase chain reaction (PCR) amplification system and a single round of hybridization. Additionally, the incorporation of ααα^anti−4.2^, ααα^anti−3.7^, ^--FIL^ deletion, and^--THAI^ deletion enhances the assay’s capabilities. Developed to concurrently identify 10 types of α-thalassemia mutations, the updated M-PCR/RDB III assay showcases enhanced effectiveness and diagnostic accuracy. This research delineates the evolution and diagnostic efficacy of the M-PCR/RDB III assay in patients originating from southern China.

## 2 Patients and methods

### 2.1 Study population

This study collected samples from patients with thalassemia and normal controls who had previously been genotyped using the reference methods (described in the Reference Methods) between April 2021 and March 2024 from the People’s Hospital of Guangxi Zhuang Autonomous Region, Zhujiang Hospital of Southern Medical University, and Xiangya Reproductive and Genetic Hospital of Central South University. Approval for this study was obtained from the Ethics Committees of the three hospitals, with the approval numbers 2020-51, 2021-SJ-001-04, and SJ2023002, respectively. The patients with thalassemia were deliberately chosen, while the healthy controls were randomly selected from routine check-up volunteers. These samples were used to evaluate the specificity and accuracy of the newly developed assay in a double-blind manner. Written consent was obtained from the patients or their guardians, as well as from the healthy volunteers, for this investigation.

The study also received approval from the Ethics Committees of People’s Hospital of Yangjiang (No.2023003). The study adheres to the STROBE guidelines (von Elm et al., 2007). Patient information was de-identified to ensure that no patient’s identity could be disclosed in any manner.

### 2.2 DNA extraction

Genomic DNA extracted from peripheral blood leukocytes of the study participants was obtained using a DNA Prep Kit (Guangdong Hybribio Limited Corporation, Chaozhou, Guangdong Province, China). The DNA concentration was assessed using a NanoDrop™ One/One C Microvolume UV-Vis Spectrophotometer (Thermo Fisher Scientific, Rockford, IL, United States) at a wavelength of 260 nm, with DNA purity evaluated based on the 260/280 nm ratio. These DNA samples were utilized for subsequent PCR analysis. The clinical test (reference methods) and validation test (M-PCR/RDB III) were conducted in the three aforementioned hospitals.

### 2.3 Design of primers and probes

**FIGURE 1:**
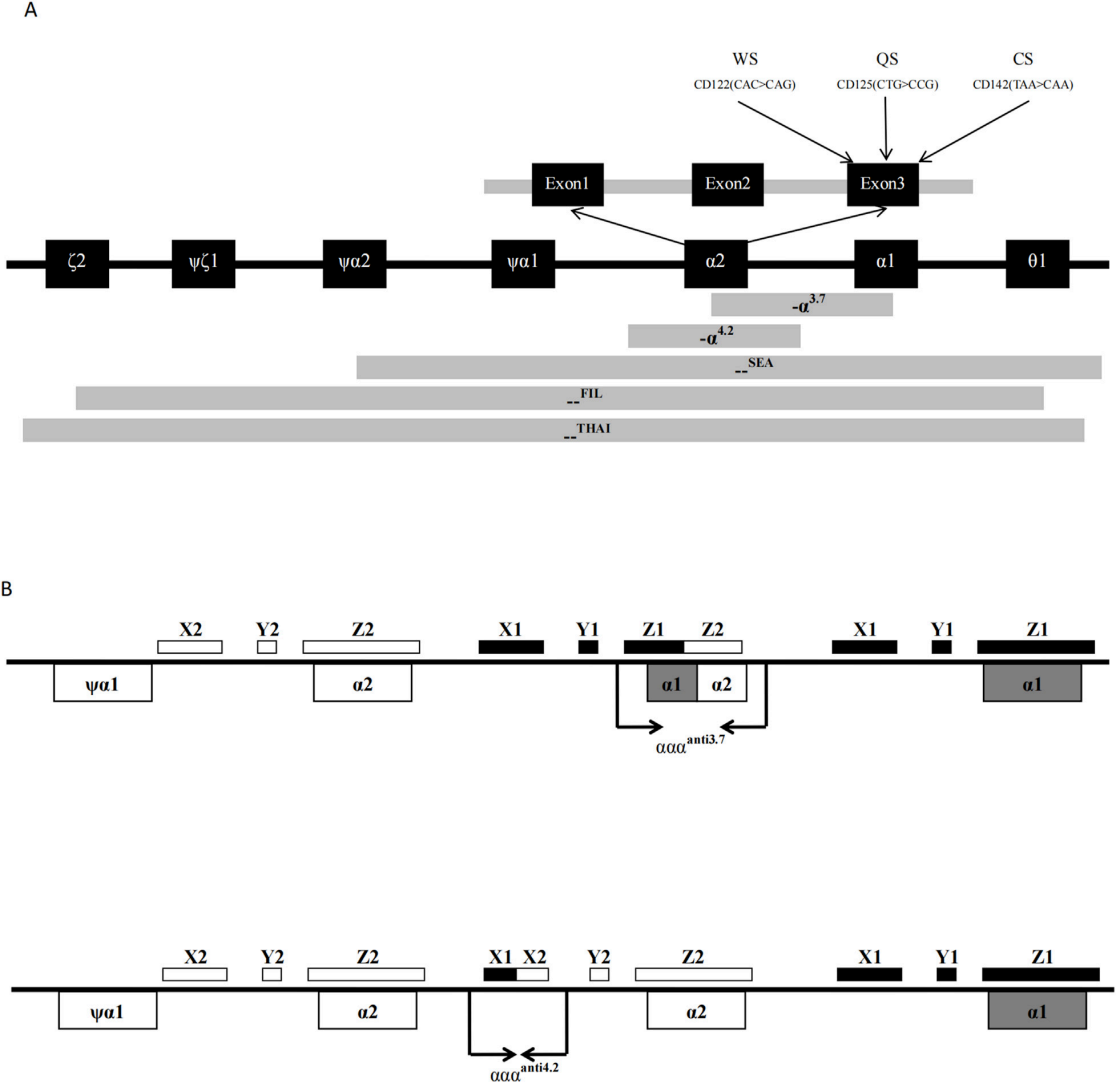
Schematic representation of the 6 kinds of common α-thalassemia allele **(A)** and 4 kinds of rare α-thalassemia allele; **(B)** location in the α-gene cluster.

**FIGURE 2:**
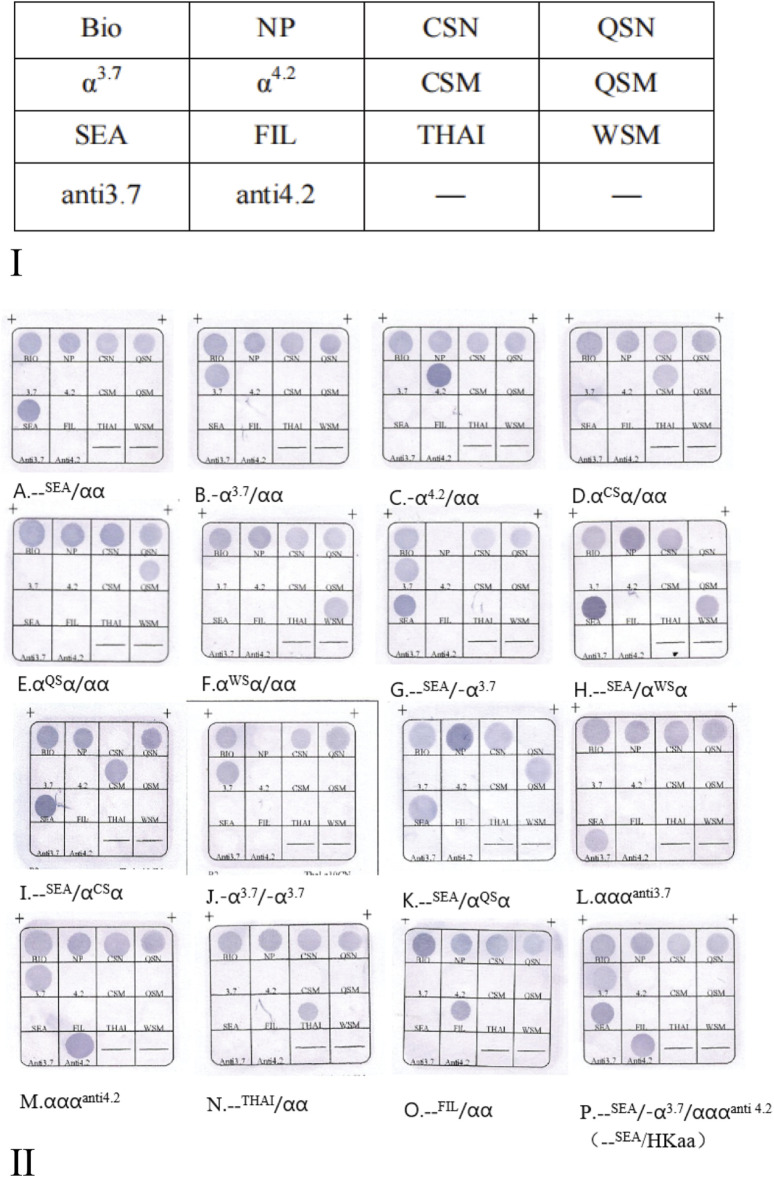
**(Ⅰ)** The probes location in the gene chip. Note: N, Normal control; M, Mutation. NP indicates a fragment for α2 gene, as the control for --^SEA^, -α^3.7^, and -α^4.2^, --^FIL^ and--^THAI^; QSN is the control for QSM and WSM, CSN is the control for CSM, respectively. ααα^anti3.7^ (referred to as anti3.7) and ααα^anti4.2^ (referred to as anti4.2) have no normal controls; Bio refers to biotin points used to monitor the hybridization process. **(Ⅱ)** Hybridization results of M-PCR/RDB Ⅲ assay. (A. --^SEA^/αα; B. -α^3.7^/αα; C. -α^4.2^/αα; D. α^CS^α/αα; E. α^QS^α/αα; F. α^WS^α/αα; G. --^SEA^/-α^3.7^; H. --^SEA^/α^WS^α; I. --^SEA^/α^CS^α; J. -α^3.7^/-α^3.7^; K. --^SEA^/α^QS^α; L.αα/ααα^anti3.7^; M. αα/ααα^anti4.2^; N. --^Thai^/αα; O. --^Fil^/αα; P. –^SEA^/-α^3.7^ααα^anti4.2^ (HKαα/--^SEA^).

The thalassemia detection kit was designed and made by Guangzhou Hybribio Medicine Science and Technology Corporation. The detection kit included two PCR reaction systems as follows. Three sets of primers of the M-PCR assay were designed to amplify three α-thalassemia deletions (the Southeast Asian [--^SEA^], the rightward deletion (-α^3.7^) and the leftward deletion (-α^4.2^)] on chromosome 16; Three sets of primers of the M-PCR assay were designed to amplify the three common α-globin gene mutations: Hb Constant Spring [Hb CS (α^CS^α) *HBA2*: c.427T > C], Hb Quong Sze [Hb QS (α^QS^α), *HBA2*: c.377T > C], Hb Westmead [Hb WS (α^WS^α), *HBA2*: c.369C > G]; One 1800 base pair (bp) fragment of α-2 globin gene was amplified as a normal control (marked NP on the hybridization membrane). Four sets of primers of the M-PCR assay were designed to amplify four rare variants ααα^anti−4.2^, ααα^anti−3.7^, ^--FIL^ deletion and^--THAI^ deletion, respectively. Oligo 6.31 (Molecular Biology Insights, Colorado Springs, CO, United States) software was used to design the primers and probes. Schematic representation of the ten types of α-thalassemia allele location in the α-gene cluster is shown in Figure 1. All probes were immobilized on a nylon membrane. Their localization in the membrane is shown in Figure 2I. The detailed information of the primers and probes are presented in Supplementary Tables S1, S2.

### 2.4 Multiplex PCR amplification

The assay was conducted following the manufacturer’s protocol (Guangzhou Hybribio Medicine Science and Technology Corporation, Guangzhou, China). In brief, α-thalassemia PCRs were amplified in two tubes. The A group included the detected genotypes: ^--SEA^, -α^3.7^, -α^4.2^, α^CS^, α^QS^, α^WS^; while the B group included the detected genotypes: ^--FIL^, ^--THAI^, ααα^anti3.7^, and ααα^anti4.2^. PCRs were carried out using a PCR appliance (GeneTouch; BIOER, Hangzhou, China), with a reaction volume of 50 μL comprising 6 μL of DNA template, 0.5 μL DNA polymerase, and 43.5 μL PCR MIX (100 μmol/L Primer MIX: 1.25 μL, 25 mmol/L MgCl_2_: 3 μL, 10 × PCR buffer: 5 μL, 5 × PCR enhancer: 7 μL, 25 mmol/L dNTPs: 0.8 μL, H_2_O: 26.45 μL). The cycling program included an initial denaturation at 95°C for 15 min, followed by 35 cycles consisting of denaturation at 98°C for 40 s, annealing at 64°C for 70 s, elongation at 72°C for 150 s, and a final elongation step at 72°C for 5 min. Subsequently, the amplicons from the reaction system (two tubes) were denatured and subjected to hybridization.

### 2.5 Flow-through hybridization

Hybridization reactions were performed using α thalassemia gene diagnostic kit and flow-through hybridization kit (Guangzhou Hybribio Medicine Science and Technology Corporation, Guangzhou, China), the reaction and hybridization condition was the same as described previously (Lin et al., 2012; Liang et al., 2022). The assay utilized flow-through hybridization technology (HB2012A; Guangdong Hybribio Limited Corporation). After hybridization, a blue-purple precipitate at the probe dot could be discerned. The results were interpreted by direct visualization.

### 2.6 Reference methods

For the reference methods, all thalassemia alleles were characterized using a combination of techniques, including M-PCR/RDB Ⅱ,M-PCR/RDB I, Sanger’s sequencing, and a two-round nested PCR strategy. The analysis involved three types of α-thalassemia deletions (^--SEA^, -α^3.7^, and -α^4.2^) and three types of α-thalassemia mutations (Hb CS, Hb WS, and Hb QS) using M-PCR/RDB I or M-PCR/RDB II (Guangdong Hybribio Limited Corporation) (Lin et al., 2012; Liang et al., 2022). Additionally, a two-round nested PCR strategy was implemented to detect the potential presence of HK αα, a rare α-thalassemia mutation reported in southern Chinese subjects (Wu et al., 2015). The^--FIL^ deletion and^--THAI^ deletion were identified using gap-PCR, as previously described (Eng et al., 2000). Furthermore, the testing for ααα^anti−3.7^ and ααα^anti−4.2^ triplications followed established protocols (Wang et al., 2003). Finally, ^--FIL^ deletion, ^--THAI^ deletion, ααα^anti−3.7^, and ααα^anti−4.2^ were subsequently validated through Sanger sequencing.

### 2.7 Statistical analyses

All statistical analyses were conducted using the SPSS^®^ statistical package, version 16.0 (SPSS Inc., Chicago, IL, United States), on Windows^®^. The agreement between M-PCR/RDB III and the reference methods was assessed using kappa statistics and McNemar’s χ -test. Statistical significance was defined as a *P*-value <0.05.

## 3 Results

This study collected samples from 1,148 unrelated participants, including 810 patients with thalassemia and 338 healthy control subjects as follows: People’s Hospital of Guangxi Zhuang Autonomous Region (380 cases), Zhujiang Hospital of Southern Medical University (385 cases), and Xiangya Reproductive and Genetic Hospital of Central South University (383 cases). The detection panel included common^--SEA^, -α^3.7^, -α^4.2^, α^CS^, α^QS^, α^WS^, and rare variants ααα^anti−4.2^, ααα^anti−3.7^, ^--FIL^ deletion, and^--THAI^ deletion in Chinese.

In a verification test, a total of 1,148 genomic DNA pre-characterized samples (810 patients with thalassemia and 338 healthy control subjects) were analyzed with the M-PCR/RDB Ⅲ assay. Valid hybridization results were obtained for 1,147 samples, with one case (thalassemia carrier) being excluded from the study due to the poor quality of DNA. The new kit successfully diagnosed 809 patients with thalassemia and 338 healthy control subjects. The hybridization results of the M-PCR/RDB Ⅲ assay are presented in Supplementary Table S3, which included α heterozygous thalassemia, α homozygous thalassemia and α compound heterozygous thalassemia (Figure 2Ⅱ).

The agreement between the M-PCR/RDB Ⅲ assay and the reference methods was found to be in absolute concordance (kappa = 1, *P* < 0.001) for detecting the three α-globin deletions (^--SEA^, -α^3.7^, -α^4.2^) (Figure 2ⅡA–C) and three α-globin mutations (α^CS^, α^QS^, α^WS^) (Figure 2ⅡD–F). Additionally, rare variants including ααα^anti 3.7^ (n = 25) (Figure 2ⅡL), ααα^anti 4.2^ (n = 24) (Figure 2ⅡM), ^--THAI^ (n = 12) (Figure 2ⅡN), and^--FIL^ (n = 4) (Figure 2ⅡO) were successfully identified using the M-PCR/RDB Ⅲ assay with 100% concordance with the reference methods (Supplementary Table S3; Figure 2Ⅱ).

The current study could also identify compound combinations such as--^SEA^/-α^3.7^(Figure 2ⅡG), --^SEA^/α^WS^α (Figure 2ⅡH), --^SEA^/α^CS^α (Figure 2ⅡI), -α^3.7^/-α^3.7^(Figure 2ⅡJ), --^SEA^/α^QS^α (Figure 2ⅡK), -α^3.7^/ααα^anti 4.2^ (n = 7), ^--SEA^/-α^3.7^ααα^anti4.2^ (HKαα/--^SEA^, n = 2) (Figure 2ⅡP), -α^3.7^/ααα^anti3.7^(n = 1), -α^3.7^/α^CS^α/ααα^anti 4.2^(n = 1), and^--SEA^/ααα^anti 3.7^(n = 1).

For cases with^--SEA^/-α^3.7^ααα^anti4.2^ (Figure 2ⅡP) genotypes, where one chromosome has been deleted (^--SEA^ deletion), the -α^3.7^ and ααα^anti4.2^ alleles are expected to be on the same chromosome. We can confirm that the genotype is HKαα/^--SEA^.

## 4 Discussion

Thalassemia is a prevalent genetic disease in southern China, significantly impacting public health in these areas with high prevalence. While a screening test involving a complete blood count, hemoglobin quantification via capillary electrophoresis and/or high performance liquid chromatography, may not identify all thalassemia subtypes, laboratory diagnosis necessitates molecular analysis. With advancements in molecular diagnostics, genetic diagnosis of thalassemia can be easily achieved through gap-PCR and PCR-RDB of the affected globin genes. These methods have demonstrated precision and sensitivity in identifying thalassemia genotypes, leading to their widespread adoption in clinical settings for the molecular diagnosis of thalassemia (Lin et al., 2012; Liang et al., 2022; Wang et al., 2003). However, despite their efficacy, these assays were unable to distinguish relatively rare α variants, such as ^anti−4.2, anti−3.7, --FIL^ deletion, and^--THAI^ deletion (Lin et al., 2012; Liang et al., 2022).

The enhanced M-PCR/RDB Ⅲ, as described, offers a more comprehensive coverage compared to the previously developed M-PCR/RDB I/M-PCR/RDB Ⅱ and other existing clinical methods. It successfully detects rare variants such as ^anti−4.2^, ^anti−3.7^, ^--FIL^ deletion, and^--THAI^ deletion. Typically, α-globin gene deletions are diagnosed using gap-PCR, which can be time-consuming due to post-PCR work (electrophoresis). The improved M-PCR/RDB Ⅲ simplifies and expands the analysis panel.

Our new kit has the capability to identify intricate and rare α variant combinations, including -α^3.7^/ααα^anti−4.2^, ^--SEA^/-α^3.7^ααα^anti−4.2^, -α^3.7^/ααα^anti−4.2^, ^--SEA^/ααα^anti−3.7^, and -α^3.7^/α^CS^ααα^anti−4.2^. However, it is noted that the new M-PCR/RDB Ⅲ assay does have a specific limitation. A rare α-thalassemia variant, the HKαα genotype (-α^3.7^/ααα^anti−4.2^), previously reported in China (Wu et al., 2015), can only be detected when it is concurrent with^--SEA^ (as seen in an individual with the--^SEA^/-α^3.7^ααα^anti−4.2^ genotype). In cases where an individual is identified with -α^3.7^ and ααα^anti−4.2^ using the M-PCR/RDB Ⅲ, certainty regarding their location on the same allele (HKαα) cannot be established.

Hong Kong type α-thalassemia (HKαα) is a recombinant gene formed by non-homologous recombination of the α-globin gene, resulting in a gene that contains both the -α^3.7^ and ααα^anti4.2^ segments, with two functional α-globin genes. Individuals with HKαα/^--SEA^, due to the presence of a functional α2 gene, have a hematological phenotype similar to that of^--SEA^ heterozygotes, indicating a mild form of α-thalassemia (Wu et al., 2015). This suggests that if one parent is HKαα/αα and the other is a^--SEA^ heterozygote, prenatal diagnosis is not necessary. However, if a mistaken diagnosis labels HKαα/αα as a -α^3.7^ heterozygote and the other parent is a^--SEA^ heterozygote, there is a 25% chance of the offspring having Hb H (intermediate α-thalassemia), prompting the need for informed consent and allowing the pregnant woman to choose whether to undergo prenatal diagnosis. This increases the difficulty of clinical genetic counseling and unnecessarily adds anxiety to the pregnant woman and her family. Therefore, accurate screening and differentiation between -α^3.7^ and HKαα have important clinical significance. A previous study conducted HKαα genotype diagnosis on 507 samples, identified as -α^3.7^/αα through Gap-PCR gene testing. Subsequent nested PCR analysis revealed that 7.27% of the samples had the HKαα genotype (Zhang et al., 2019). In another study on Chinese carriers of silent deletional α-thalassemia, the frequencies of the HKαα and anti-HKαα alleles were 2.27% and 0.35% in -α^3.7^ and -α^4.2^ carriers, respectively (Zhong et al., 2018).

The α-globin triplication is the result of unequal exchange of homologous sequences in the α-globin gene cluster of chromosome 16 during meiotic pairing. The homologous region of the α-globin gene cluster includes X, Y, and Z boxes, with two Z homologous boxes spaced 3.7 kb apart and two X homologous boxes spaced 4.2 kb apart. Recombination at the Z homologous boxes can lead to a 3.7 kb deletion on one chromosome (-α^3.7^), forming the α-globin triplication (ααα^anti3.7^) on the other chromosome. Recombination at the X homologous boxes can result in a 4.2 kb deletion (-α^4.2^) and another type of α-globin triplication (ααα^anti4.2^) (Wang et al., 2003).

There are relevant literature reports on the prevalence of α-globin triplication in populations both domestically and internationally. In 2018, Iranian scholar Seyedeh (Abedini et al., 2018) conducted α-globin triplication detection on 4,010 individuals from different provinces, revealing a carrier rate of 1.7% in the population. In China, a study on 1,169 newborn umbilical cord blood samples suggested a carrier rate of 1.2% for two types of α-globin triplication (Xie et al., 2015). Research data from 20,222 individuals across five southern provinces of China conducted in 2017 revealed a high carrier rate of α-globin triplication at 1.67% (Shang et al., 2017).

In terms of clinical phenotype and hematological changes, most current views still suggest that the phenotype becomes more severe after the compound of β-thalassemia mutations with α-globin triplication. This is mainly due to the increased copy number of α-globin genes leading to an imbalance in the ratio of α-globin peptide chains to β-globin peptide chains, resulting in moderate to severe anemia, skin pallor, and hepatosplenomegaly, as well as hematological characteristic changes in most thalassemia patients (Moosavi et al., 2011), some of whom require intermittent blood transfusions for treatment.

In this scenario, our M-PCR/RDB III assay was especially beneficial for a couple where one partner carries a β-thalassemia mutation, necessitating α-globin triplication analysis for the other individual. If α-globin triplication is confirmed in the latter, they should be notified, and if desired, prenatal diagnosis could be pursued. In conclusion, this enhanced multiplex-PCR-based RDB assay has the potential to greatly enhance the screening panel for both common and rare genotypes of α-thalassemia in southern China.

## Data Availability

The raw data supporting the conclusion of this article will be made available by the authors, without undue reservation.
