# Supplementary material for: Development and clinical validation of a novel detection kit for α-thalassemia in southern Chinese
Source: Front Genet. 2024 Sep 5;15:1457248. doi: 10.3389/fgene.2024.1457248 (PMC11410688; doi:10.3389/fgene.2024.1457248)
Supplement: Supplementary file 1 [file Table1.DOCX]

Table S1. Detailed information for the primers in the PCR reaction system.

| Primers | Sequence(5'→3') | Position |
| --- | --- | --- |
| HBA-1F (forward) | 5'-CCTCGCCAAGTCCAC-3' | Hg38 chr16: 171885-171899 |
| HBA-1R (reverse) | 5'-ACCAGGAAGGGCCGG-3' | Hg38 chr16: 173669-173683 |
| HBA-2F (forward) | 5'-CCTCGCCAAGTCCAC-3' | Hg38 chr16: 171885-171899 |
| HBA-2R (reverse) | 5'-AGCACTCTAGGGTCCAG-3' | Hg38 chr16: 177701-177717 |
| HBA-3F (forward) | 5'-TTTACCCATGTGGTGCC-3' | Hg38 chr16: 169270-169286 |
| HBA-3R (reverse) | 5'-CGTTGGATCTTCTCATTTC-3' | Hg38 chr16: 175122-175140 |
| HBA-4F (forward) | 5'-ATCTGGGCTCTGTGTTC-3' | Hg38 chr16: 165259-165275 |
| HBA-4R (reverse) | 5'-CCCACGTTGTGTTCATG-3' | Hg38 chr16: 185891-185907 |
| HBA-5F (forward) | 5'-ACCTCCATTCTCCAACC-3' | Hg38 chr16: 175510-175526 |
| HBA-5R (reverse) | 5'-AGGGCCCGTTGGGAG-3' | Hg38 chr16: 173639-173653 |
| HBA-6F (forward) | 5'-TTGCACCGGCCCTTCC-3' | Hg38 chr16: 173664-173679 |
| HBA-6R (reverse) | 5'-ACTGGCTGAAAGGGATG-3' | Hg38 chr16: 171097-171113 |
| HBA-7F (forward) | 5'-AGGCTATGGTGGGCAA-3' | Hg38 chr16: 150202-150217 |
| HBA-7R (reverse) | 5'-AACCTTTATCTGCCACATGTA-3' | Hg38 chr16: 182640-182660 |
| HBA-8F (forward) | 5'-CCATTCCTCAGCGTGGG-3' | Hg38 chr16: 148731-148747 |
| HBA-8R (reverse) | 5'-GAGTGCAAATTCCCCTG-3' | Hg38 chr16: 183428-183444 |

Note: HBA-1F/HBA-1R：The primers for α^CS^, α^QS^, and α^WS^; HBA-2F/HBA-2R：The primers for -α^3.7^; HBA-3F/HBA-3R：The primers for -α^4.2^; HBA-4F/HBA-4R：The primers for ^–SEA^; HBA-5F/HBA-5R：The primers for ααα^anti3.7^; HBA-6F/HBA-6R：The primers for ααα^anti4.2^; HBA-7F/HBA-7R：The primers for ^–FIL^; HBA-8F/HBA-8R：The primers for ^–THAI^.

The amplification is divided into two tubes. Group A includes the following genotypes: ^--SEA^, -α^3.7^, -α^4.2^, α^CS^, α^QS^, and α^WS^; Group B includes the following genotypes: ^--FIL^, ^--THAI^, ααα^anti3.7^, and ααα^anti4.2^.
