# Supplementary material for: Development and clinical validation of a novel detection kit for α-thalassemia in southern Chinese
Source: Front Genet. 2024 Sep 5;15:1457248. doi: 10.3389/fgene.2024.1457248 (PMC11410688; doi:10.3389/fgene.2024.1457248)
Supplement: Supplementary file 2 [file Table2.DOCX]

Table S2. Detailed information for the probes in the thalassemia gene chip.

| Probe | Sequence(5'→3') | Position |
| --- | --- | --- |
| NP | AGGAGGAACGGCTA | Hg38 chr16: 173614-173627 |
| CSN | CCAGCTTAACGGTA | Hg38 chr16: 173592-173605 |
| CSM | CAGCTTGACGGTA | Hg38 chr16: 173592-173604 |
| QSN | GTCCAGGGAGGCG | Hg38 chr16: 173540-173552 |
| QSM | CCCCGGACAAGTT | Hg38 chr16: 173545-173557 |
| WSM | GGAGGCCTGCACC | Hg38 chr16: 173534-173546 |
| -α3.7 | GAAAAAACTCAGGCA | Hg38 chr16: 177532-177546 |
| -α4.2 | AGCGAAACTCCGC | Hg38 chr16: 175021-175033 |
| --SEA | CAGCCTCCCGACTAG | Hg38 chr16: 185000-185014 |
| anti 3.7 | ATTCAACCTCCTCTG | Hg38 chr16: 175756-175770 |
| anti 4.2 | CTCCTCACACCCAC | Hg38 chr16: 170908-170921 |
| --FIL | CCCGACTCCGCTAA | Hg38 chr16: 150414-150427 |
| --THAI | CTCAGCCCACTTG | Hg38 chr16: 183321-183333 |
