# Supplementary material for: Development and clinical validation of a novel detection kit for α-thalassemia in southern Chinese
Source: Front Genet. 2024 Sep 5;15:1457248. doi: 10.3389/fgene.2024.1457248 (PMC11410688; doi:10.3389/fgene.2024.1457248)
Supplement: Supplementary file 3 [file Table3.DOCX]

Table S3. The results of a verification test analyzed genomic DNA pre-characterized samples from 809 patients with thalassemia and using the novel polymerase chain reaction/reverse dot blot assay **Ⅲ**.

| α-Thalassemia | Cases (n) |
| --- | --- |
| ^--SEA^/αα | 210 |
| -α^3.7^/αα | 145 |
| -α^4.2^/αα | 80 |
| α^CS^α/αα | 75 |
| α^QS^α/αα | 70 |
| α^WS^α/αα | 44 |
| ααα^anti 3.7^ | 25 |
| ααα^anti 4.2^ | 24 |
| ^--SEA^/-α^3.7^ | 24 |
| ^--SEA^/α^WS^α | 23 |
| ^--SEA^/α^CS^α | 18 |
| ^--SEA^/-α^4.2^ | 12 |
| ^--THAI^/αα | 12 |
| -α^3.7^/ααα^anti 4.2^ | 7 |
| -α^3.7^/α^WS^α | 6 |
| -α^3.7^/-α^3.7^ | 5 |
| -α^3.7^/α^CS^α | 5 |
| -α^3.7^/-α^4.2^ | 5 |
| ^--SEA^/α^QS^α | 4 |
| ^--FIL^/αα | 4 |
| -α^4.2^/α^CS^α | 3 |
| ^--SEA^/-α3.7/ααα^anti 4.2^ | 2 |
| -α^4.2^/α^WS^α | 2 |
| -α^3.7^/ααα^anti 3.7^ | 1 |
| -α^3.7^/α^CS^α/ααα^anti 4.2^ | 1 |
| -α^4.2^/-α^4.2^ | 1 |
| ^--SEA^/ααα^anti 3.7^  Total | 1  809 |
